# Comparative analysis of pepper and tomato reveals euchromatin expansion of pepper genome caused by differential accumulation of *Ty3/Gypsy*-like elements

**DOI:** 10.1186/1471-2164-12-85

**Published:** 2011-01-29

**Authors:** Minkyu Park, SungHwan Jo, Jin-Kyung Kwon, Jongsun Park, Jong Hwa Ahn, Seungill Kim, Yong-Hwan Lee, Tae-Jin Yang, Cheol-Goo Hur, Byoung-Cheorl Kang, Byung-Dong Kim, Doil Choi

**Affiliations:** 1 Interdisciplinary Program in Agriculture Biotechnology, Seoul National University, Seoul 151-921, Korea; 2 Plant Genomics and Breeding Institute, Seoul National University, Seoul 151-921, Korea; 3 Seeders Inc., Daejeon, Korea; 4 Bioinformatics Research Center, KRIBB, Daejeon, 305-806, Korea; 5 Department of Plant Science, Seoul National University, Seoul, 151-921, Korea; 6 Fungal Bioinformatics Laboratory, Seoul National University, Seoul 151-921, Korea; 7 Center for Fungal Pathogenesis, Seoul National University, Seoul 151-921, Korea; 8 Department of Agricultural Biotechnology, Seoul National University, Seoul 151-921, Korea

## Abstract

**Background:**

Among the *Solanaceae *plants, the pepper genome is three times larger than that of tomato. Although the gene repertoire and gene order of both species are well conserved, the cause of the genome-size difference is not known. To determine the causes for the expansion of pepper euchromatic regions, we compared the pepper genome to that of tomato.

**Results:**

For sequence-level analysis, we generated 35.6 Mb of pepper genomic sequences from euchromatin enriched 1,245 pepper BAC clones. The comparative analysis of orthologous gene-rich regions between both species revealed insertion of transposons exclusively in the pepper sequences, maintaining the gene order and content. The most common type of the transposon found was the LTR retrotransposon. Phylogenetic comparison of the LTR retrotransposons revealed that two groups of *Ty3/Gypsy*-like elements (Tat and Athila) were overly accumulated in the pepper genome. The FISH analysis of the pepper Tat elements showed a random distribution in heterochromatic and euchromatic regions, whereas the tomato Tat elements showed heterochromatin-preferential accumulation.

**Conclusions:**

Compared to tomato pepper euchromatin doubled its size by differential accumulation of a specific group of *Ty3/Gypsy*-like elements. Our results could provide an insight on the mechanism of genome evolution in the *Solanaceae *family.

## Background

The *Solanaceae *is an unusually divergent family consisting of approximately 90 genera and 3,000-4,000 species [1]. Members of the *Solanaceae *have evolved into extremely divergent forms, ranging from trees to annual herbs, and they occupy diverse habitats ranging from deserts to aquatic areas [1]. Such hyper-diversity in one family makes it useful to study plant adaptation and diversification. Despite this diversity, all Solanaceous species evolved during the last 40 million years [2]. Furthermore, almost all members share the same chromosome number (x = 12) [2].

To date, diversity within the *Solanaceae *has been studied by comparative genome analyses using common genetic markers. As a result, we know that the *Solanaceae *genomes have undergone relatively small numbers of chromosomal rearrangements (*e.g.*, about 5 rearrangements between potato and tomato and about 30 rearrangements between pepper and tomato), maintaining well-conserved gene content and order [3-8]. The conservation of the *Solanaceae *genic region was also identified by the comparison of a syntenic segment in eggplant, pepper, petunia and tomato [7].

Despite such conservation, the genome sizes of the *Solanaceae *family members are diverse. For example, the genome size of the *Solanum tuberosum *(potato) is 840 Mb, *S. lycopersicum *(tomato) 950 Mb, *Petunia hybrida *(petunia) 1200 Mb, and *Capsicum annuum *(pepper) 2700 Mb. However, the genetic analyses conducted to date were not successful at explaining genome size diversity due to limitations in the genetic markers. Hence, a sequence-level analysis to investigate the cause of the genome size diversity is required.

Among the Solanaceous species, pepper and tomato show strong advantages for the study of genome size difference because of following reasons. First, the genome size of pepper is three times larger than that of tomato. Second, the duplication of the whole genome did not occur during the evolution of both species [8]. Third, although pepper and tomato show large size differences in their genomes, their speciation is estimated to have occurred recently (approximately 16.2-22.2 million years ago) [7], which makes them not as closely related as potato and tomato, but more closely related than tobacco and tomato within the *Solanaceae *family [9]. Therefore, the investigation of genome diversity between pepper and tomato can represent the general trend of genome diversification among Solanaceous members that have not undergone the whole genome duplication.

To date, most studies related to the pepper genome have been carried out by generating genetic maps [6,10-14]. In contrast, the structure of the tomato euchromatic and heterochromatic regions has been the subject of several studies through the analyses of tomato BAC sequences [15-17]. Furthermore, the tomato genome sequencing project is currently underway, with the goal of generating a reference genome in the *Solanaceae *[18-21].

As a first study concerning the expansion of the pepper genome, the present work addresses the causes behind the expansion of pepper euchromatic regions. For this purpose, 35.6 Mb of pepper sequences from 1,245 BAC clones selected from euchromatin-enriched regions were generated. Using information from the tomato genome project, 39.9 Mb BAC sequences of tomato were chosen for comparing orthologous gene-rich sequences and the constitution of repetitive elements between the pepper and tomato genomes. We used fluorescence *in situ *hybridization (FISH) to support the results. This study presents an example of the *Solanaceae *genome diversity revealing how the pepper euchromatic region was expanded.

## Results

### Sequencing of pepper BAC clones

**Figure 1:**
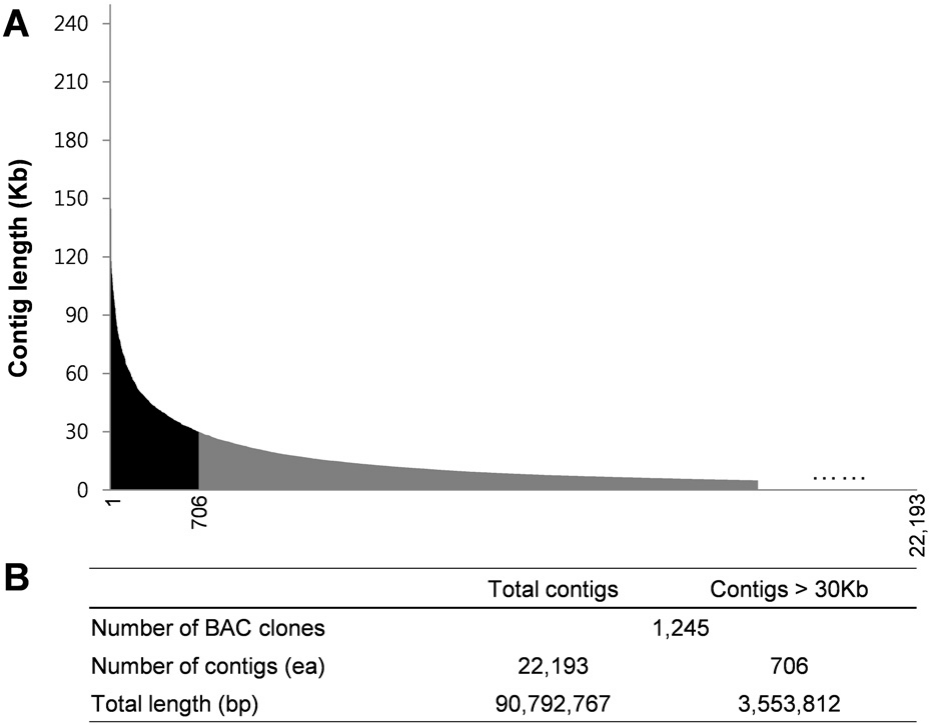
**Information about 1,245 pepper BAC sequences**. (a) Histogram of the assembled contig sizes. The contigs longer than 30 Kb are depicted by a black area and the shorter contigs are shown in gray. The contigs longer than 5 Kb are depicted in this histogram. (b) Information about contig number and total length. A total of 706 out of 22,193 contigs were longer than 30 Kb and their total length was about 35.6 Mb. This 35.6 Mb sequence was used in the analysis.

To produce the pepper sequence data representative of all pepper euchromatic regions, 1,235 pepper BAC clones of an average insert size of 130 Kb [22] were sequenced using pyrosequencing technology. To enrich the euchromatic regions, BAC clones were selected by BAC screening using labelled cDNAs derived from pepper mRNAs (extracted from flower, fruit, stem and leaf). A total of 90.8 Mb of assembled sequences was obtained from 18.22× coverage sequences generated by 454 GS FLX-Titanium (454 Life Science, Roche). To avoid the bias caused by the short contig length, we used the long contigs, whose length is over 30 Kb (total length is 34.6 Mb), in the analyses (Figure 1). In addition, ten selected pepper BAC clones containing gene-rich regions of pepper chromosome 2 were sequenced using Sanger methods, resulting in a total of 985,237 bp contig sequences (see Additional file 1). Three of the ten BAC sequences were assembled into one contig, resulting in a total of eight full-contig BAC sequences. These eight full-contig BAC sequences were used in the comparative micro-synteny analysis of pepper and tomato euchromatic regions.

### Comparison of visible genome structures

**Figure 2:**
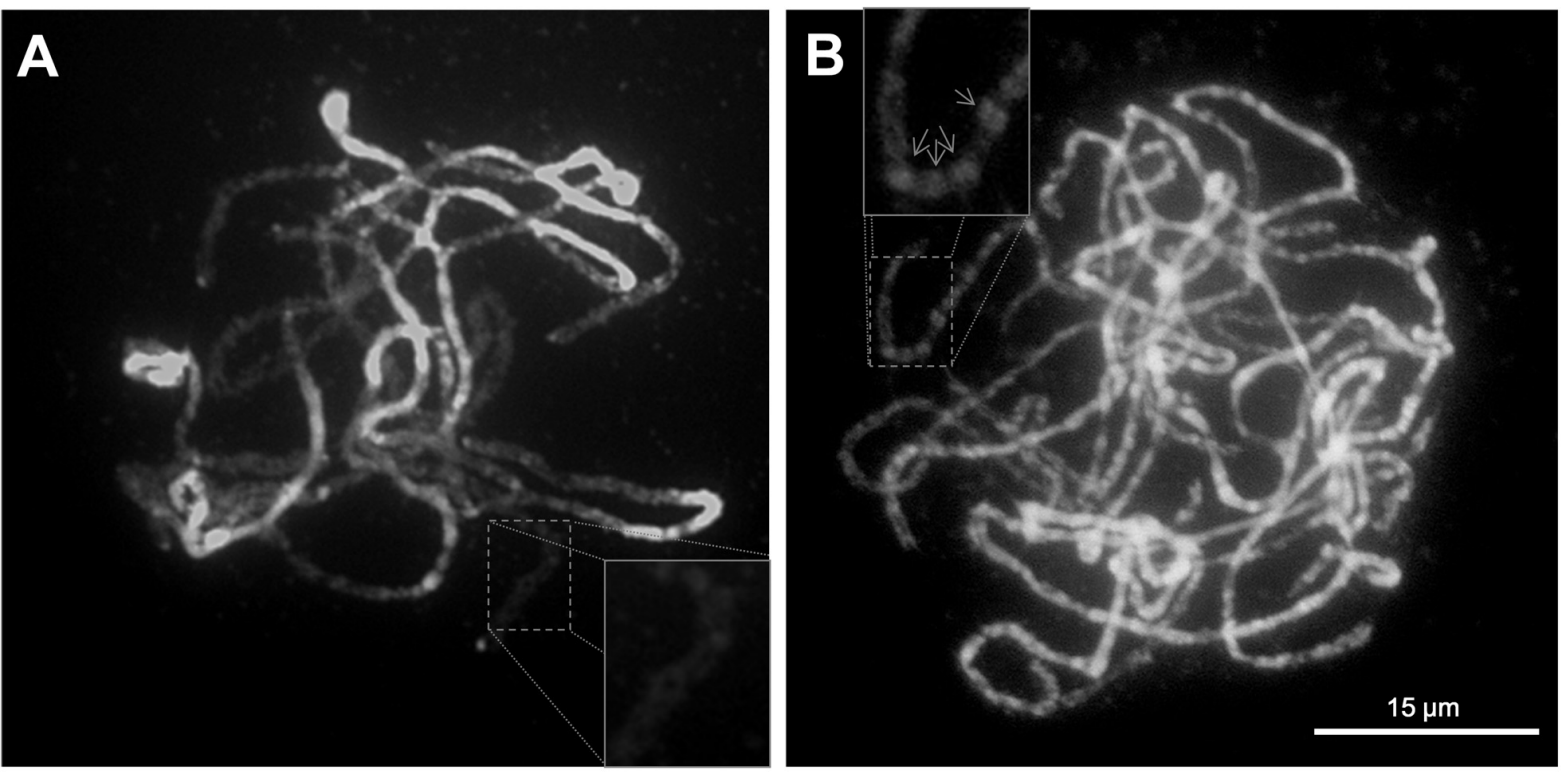
**Microscopic structures of pachytene chromosomes of tomato (a) and pepper**. **(b)**. The pachytene chromosomes were stained with DAPI and the images were converted to black and white. The heterochromatic and euchromatic regions are shown as bright and dark lines, respectively.

Prior to the comparative sequence analysis between pepper and tomato, we analyzed visible chromosome structures in pepper and tomato using pachytene chromosomes. On visual inspection, the pepper and tomato chromosomes showed differences in structure. The tomato heterochromatic regions were mainly located on the pericentromeric regions and the euchromatic regions were clearly distinct from the heterochromatin structure (Figure 2A). In contrast, the pepper pachytene chromosomes showed more extensive heterochromatic regions (Figure 2B). Furthermore, the pepper euchromatic regions were intermixed with the heterochromatin structure (Figure 2B; indicated by arrows).

### Comparison of repetitive elements in the orthologous gene-rich regions

**Figure 3:**
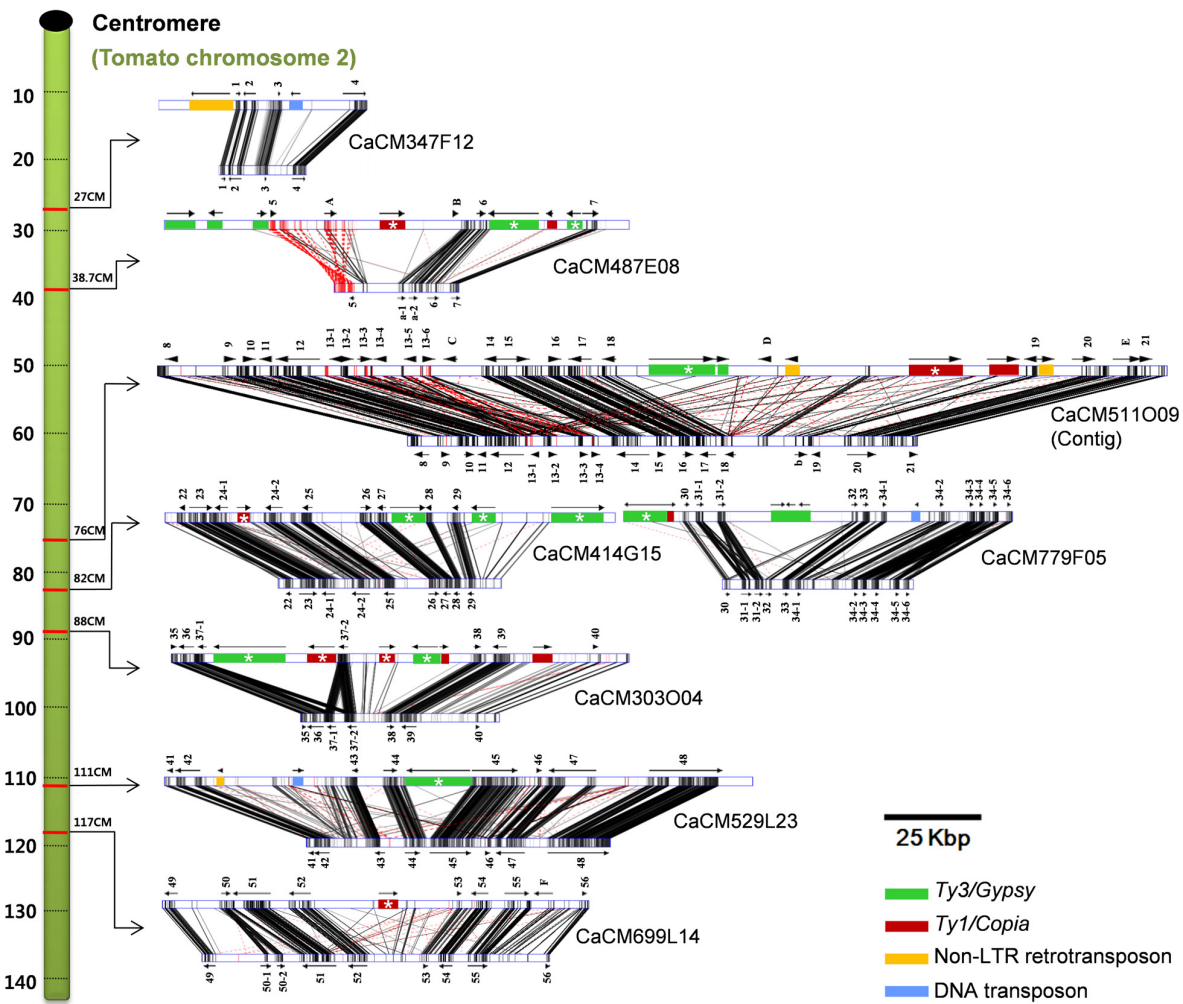
**Sequence comparisons between orthologous gene-rich regions of pepper and tomato**. The green column on the left represents the tomato chromosome 2. A black dot on the top of the green column indicates the location of the centromere. The genetic location of each orthologous sequence pair was determined on the basis of the tomato genetic map (Tomato EXPEN-2000) [23] and is indicated by a red line on the green column. Pairs of horizontal bars represent the pepper (upper) and tomato (lower) sequences. Pepper clone names are presented on the right side of each sequence pair. Highly similar regions are depicted by black lines and inverted regions by red lines. Arrows indicate predicted genes and the number sets indicate the orthologous gene sets. Letters indicate genes that have no orthologous pairs. The colored boxes indicate transposable elements. The asterisks in the colored boxes indicate the transposons that the boundary is defined. The compared sequences show many highly syntenic regions, with many insertions in the pepper sequences. For detailed information on the compared BAC sequences, see Additional file 1 and 2.

To investigate the reasons for the presence of differences in euchromatin structure between both genomes, the orthologous gene-rich sequences of pepper and tomato were compared. To compare within the same chromosome, the orthologous gene-rich sequences were selected in chromosome 2 that has no inter-chromosomal crossover between both species [8]. BAC sequences distributed over seven positions in chromosome 2 were used to avoid bias based on position within the chromosome (Figure 3 and Additional file 1). The positions of the BAC sequences were determined using genetic markers on the tomato genetic map (tomato-EXPEN 2000, http://sgn.cornell.edu/) [23]. On the basis of tomato chromosome 2, the centromere is located at the top (0 CM) of the tomato genetic map (Figure 3) [15]. Eight orthologous pepper BAC sequences of a total of 985,237 bp were compared with the tomato sequences consisting of 490,745 bp (Table 1).

**Table 1:** Statistics of the compared pepper and tomato gene-rich sequences

	Pepper	Tomato	Total (ratio)
Total length of compared sequence	985,237 bp	490,745 bp	-
Number of predicted genes	75	70	145
Total length of predicted genes	247,338 bp	195,342 bp	-
Gene density	13,136 bp/gene	7,011 bp/gene	-
Genes paired into orthologous set	69	67	136 (94%)
Genes that have no ortholog	6	3	9 (6%)
Duplicated genes	18	19	37 (25%)
Average length of coding region	1,366 bp	1,332 bp	-
Average length of intron	1,815 bp	1,459 bp	-

The comparative analysis of the orthologous gene-rich sequences revealed many insertions found exclusively in the pepper sequences. The insertions were transposable elements, and there were 35 transposable elements in the compared pepper sequences (Figure 3; colored boxes and Additional file 2). Boundary of the LTR-retrotransposons was determined in 16 elements by manual inspection (Figure 3; marked with asterisks in the colored boxes). The else transposons were found by gene prediction and repeat BLAST search in Repbase [24,25]. All of the transposable elements were found in the inter-genic regions, therefore without a disruption of the other structural genes. The insertion of the transposable elements resulted in a doubling of the pepper sequence size in comparison to that of tomato. Accordingly, the gene density was lower in pepper (13,136 bp per one gene) than in tomato (7,011 bp per one gene).

**Figure 4:**
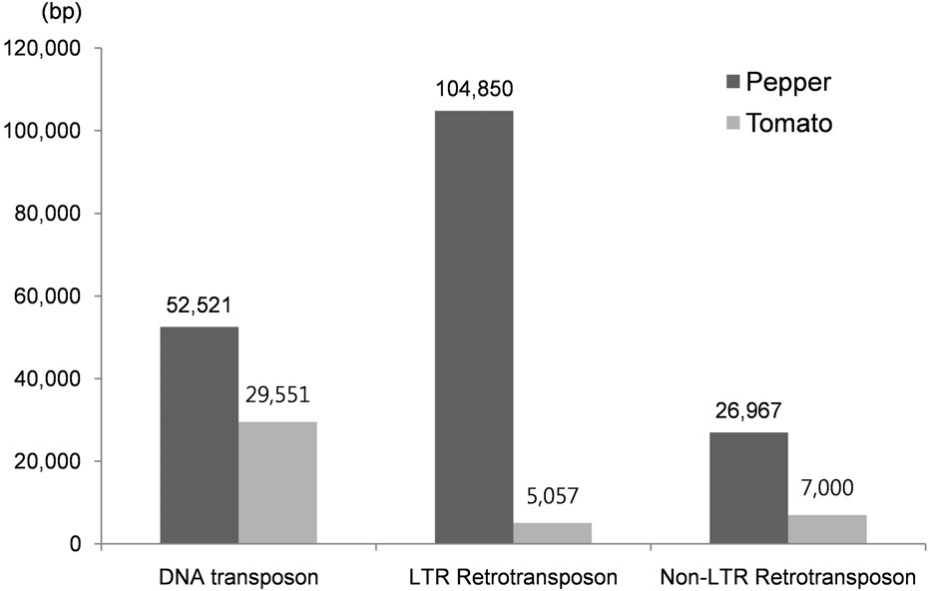
**Analysis of the compared clones for repetitive elements**. Three kinds of repetitive elements in the pepper and tomato BAC clones were compared by the total length. Among the repetitive elements, the pepper LTR retrotransposon shows the most significant difference.

To determine the most prevalent type of transposon, the composition of the repetitive elements found in the compared sequences was analyzed. By repeat BLAST search in Repbase, a total of 191,393 bp of transposon sequences were found in pepper and 44,336 bp in tomato. The repeat sequences were classified into three groups (Figure 4).

Among identified transposable elements, LTR-retrotransposon sequences were the most abundant. Most of the LTR-retrotransposons were found in the pepper sequences (Figure 4). In addition, 28 of the 35 transposable elements found in pepper sequences were identified as LTR retrotransposons. The pepper sequences contained LTR-retrotransposon sequences with a frequency approximately 22 times higher than in the tomato sequences. The other two repeat classes also presented higher proportions in pepper than in tomato. Pepper had about 1.7 times as many DNA transposons, 4 times the number of non-LTR retrotransposons (Figure 4).

According to our transposon annotation results, the total length of the transposons found was 210,341 bp (see Additional file 2). Among them, *Ty3/Gypsy*-like element was the most abundant as 134,523 bp in total length (approximately 64% of the annotated repeats), which suggests its important role in pepper euchromatin expansion. The next was *Ty1/Copia*-like elements as 55,173 bp (approximately 26% of the annotated repeats). The Non-LTR retrotransposon and DNA transposon was 12,159 bp and 5,486 bp, respectively.

### Similar gene composition between pepper and tomato

In contrast to the repetitive elements, gene constitution less affected the difference in the sequence size. In the compared sequences, a total of 145 genes were predicted excluding the transposable element genes. These included 75 pepper genes and 70 tomato genes (Table 1). The total length of the genes combined was 247,338 bp in pepper and 195,342 bp in tomato, showing a length difference of 51,996 bp. The total gene-length difference corresponded to approximately 10% of the total length difference of the compared sequences. The total gene-length difference was mainly caused by the intron-length difference. A total of 136 out of 145 genes were paired into 56 orthologous sets (see Additional file 2). In these sets the average length of the pepper coding regions was 1,366 bp, which was 34 bp longer than that of tomato (1,332 bp), whereas the average intron length in pepper was 1,815 bp, which was 356 bp longer than that of tomato (1,459 bp). Among the 56 orthologous sets, six sets (10.7%) consisted of duplicated genes. These six sets corresponded to 37 of the 145 genes (25%), of which 18 genes were in pepper and 19 in tomato (see Additional file 3). Hence, there was no remarkable bias in gene duplication number between both species (Table 1).

### Identification of LTR retrotransposons in the pepper and tomato genome sequences

The causes for the accumulation of LTR retrotransposons in the pepper euchromatic regions were investigated by comparing the overall constitution of LTR retrotransposons between pepper and tomato by phylogenetic analysis. For this analysis, reverse transcriptase (RT) sequences, which are constitutive genes in LTR retrotransposons [26], were identified from the pepper and tomato genome sequences. The RTs were classified into *Ty3/Gypsy *and *Ty1/Copia *types by BLAST search in Repbase [24,25]. A total of 155 *Ty3/Gypsy*-like and 166 *Ty1/Copia*-like tomato RTs were identified from 39.9 Mb of tomato BAC sequences (http://sgn.cornell.edu/about/tomato_sequencing.pl; downloaded in August, 2008) and 312 *Ty3/Gypsy*-like and 48 *Ty1/Copia*-like pepper RTs were found in the 35.6 Mb pepper BAC sequences. Because the tomato genome project focused on the gene-rich region, the number of heterochromatin-preferential LTR retrotransposons might be underestimated in this comparison.

### Differential accumulation of a group of *Ty3/Gypsy*-like elements

**Figure 5:**
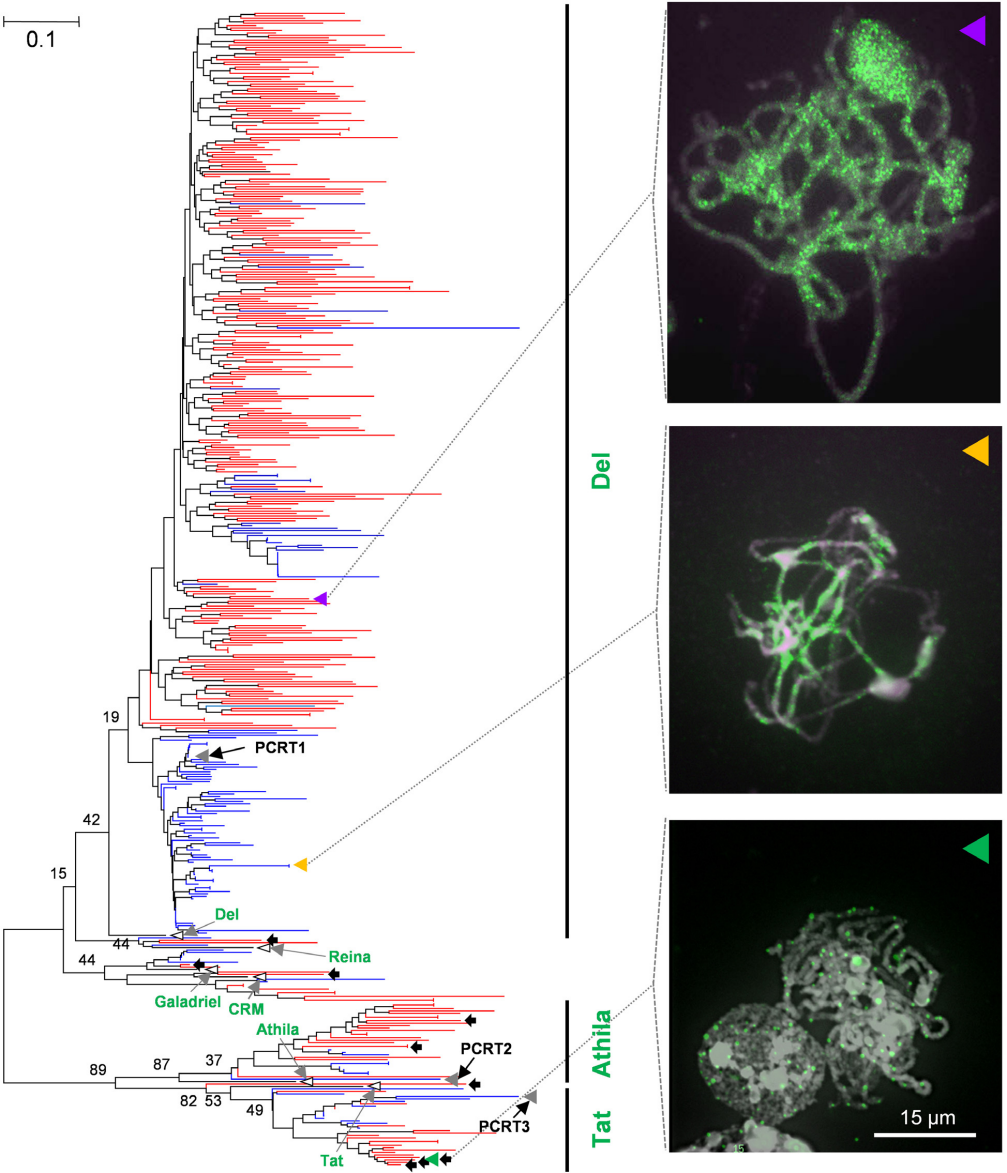
**Phylogenetic analysis of pepper and tomato *Ty3/Gypsy*-like elements**. Pepper and tomato RTs of the *Ty3/Gypsy*-like elements were used to generate the phylogenetic tree. The pepper and tomato *Ty3/Gypsy*-like elements are depicted by red and blue lines, respectively. Classified subgroups Tat, Athila and Del, are depicted by green letters. The RTs used as FISH probes are marked with triangles (purple, yellow, and green). The FISH result for each of the probes is indicated by the dotted lines (see text for details). The black arrows indicate the RTs found from the compared pepper gene-rich sequences. The empty black triangles indicate the RTs of the representative elements of each subgroup which are acquired from the GyDB. The bootstrap values were produced by a replication of 1000.

The phylogenetic tree of *Ty3/Gypsy*-like elements was generated by 312 pepper and 155 tomato RTs. A total of three subgroups were clearly identified from the phylogenetic tree (Figure 5). Each subgroup was classified on the basis of reported elements by BLAST search against GyDB (http://gydb.org/). The BLAST results with high confidence (e-value below e-40) were used for classification reference [27]. The representative elements of each subgroup which are acquired from the GyDB were also included in the phylogenetic tree. According to the classification, the three groups belonged to Tat and Athila subgroups, which belong to Athila/Tat, and to Del subgroup, which belongs to chromoviruses. Most of the *Ty3/Gypsy*-like elements were found in the three major subgroups.

The *Ty3/Gypsy*-like elements in the Del subgroup were identified as being accumulated in the pericentromeric heterochromatin. Yang *et al. *reported that the PCRT1 in the Del subgroup is a tomato *Ty3/Gypsy*-like element distributed throughout pericentromeric heterochromatin of tomato [16]. This result was consistent with our FISH result of another tomato Del element (Figure 5; indicated by yellow triangle). Furthermore, the FISH result of the pepper Del element exhibited the same distribution pattern as that of tomato (Figure 5; indicated by purple triangle). These results suggest that the Del elements constitute pericentromeric heterochromatin in both genomes, which means they do not affect euchromatin expansion.

The differences that may affect the expansion of pepper euchromatic regions were observed in the Tat and Athila subgroups. The number of pepper Tat and Athila elements was approximately twice the number in tomato (42 in pepper and 23 in tomato). According to the previous report by Yang *et al.*, PCRT2 and PCRT3 are the tomato *Ty3/Gypsy*-like elements preferentially distributed in the tomato heterochromatic regions [16]. These two elements belonged to Athila and Tat respectively, suggesting that the tomato *Ty3/Gypsy*-like elements in these groups are accumulated in heterochromatic regions. In contrast, the FISH result of the pepper Tat element showed randomly distributed signals throughout the pepper chromosomes including the euchromatic regions (Figure 5; indicated by green triangle). Furthermore, four of the nine black arrows indicating the pepper *Ty3/Gypsy*-like elements found in the compared pepper gene-rich sequences belonged to Tat. Likewise, two of the nine elements belonged to the Athila subgroup, indicating the elements in this group are also found in pepper gene-rich regions. However, the Del subgroup didn't contain any of the *Ty3/Gypsy*-like elements found in the pepper gene-rich sequences (Figure 5). These results show that, in contrast to the distribution in tomato, the pepper *Ty3/Gypsy*-like elements in the Tat and Athila subgroups are randomly inserted throughout the whole genome, including the euchromatic regions.

### Chromodomains in the *Ty3/Gypsy*-like elements

**Figure 6:**
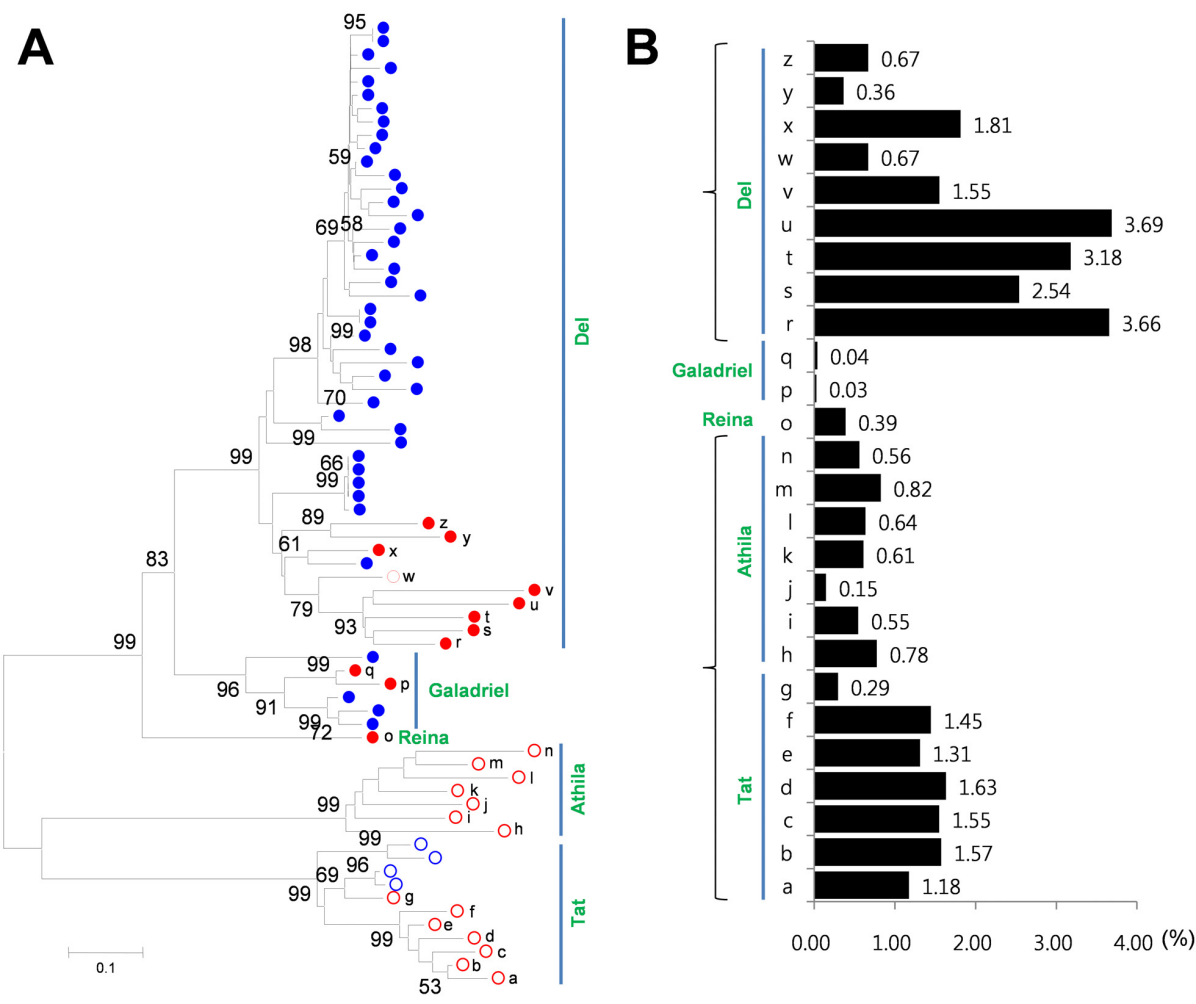
**The existence of the chromodomain and genome proportion of the intact form of the *Ty3/Gypsy-*like elements**. (a) Existence of chromodomains in the *Ty3/Gypsy-like *elements. RTs of the intact LTR retrotransposons were used in generating the phylogenetic tree. The red and blue dots indicate the pepper and tomato *Ty3/Gypsy-*like elements, respectively. The filled and empty dots indicate the existence and absence of the chromodomains, respectively. Classified types of each subgroup are depicted by green letters. The bootstrap values were produced by a replication of 1000. (b) Genome proportions of the pepper intact *Ty3/Gypsy*-like elements. The individual intact *Ty3/Gypsy*-like elements are marked by the letters 'a' to 'z' in the phylogenetic tree and graph.

A chromodomain functions to recognize the heterochromatic regions when the *Ty3/Gypsy*-like elements insert into chromosomes [28,29]. To determine the chromatin selectivity of the *Ty3/Gypsy*-like elements, the existence of the chromodomain was investigated in each group. For this analysis, 72 intact *Ty3/Gypsy*-like elements were identified from the pepper and tomato sequences to check the chromodomain (see Additional file 4). Except for the Tat and Athila elements, almost all of the other intact *Ty3/Gypsy*-like elements contained the chromodomain (Figure 6A; filled dots, Additional file 5). The existence of the chromodomain in the Del intact elements was consistent with the heterochromatin-preferential accumulation of the Del elements in both species. Likewise, the absence of the chromodomain in Tat and Athila was consistent with the random accumulation of pepper elements. However, the absence of the chromodomain was in disagreement with the anticipated heterochromatin-preferential accumulation of tomato Tat and Athila elements.

**Figure 7:**
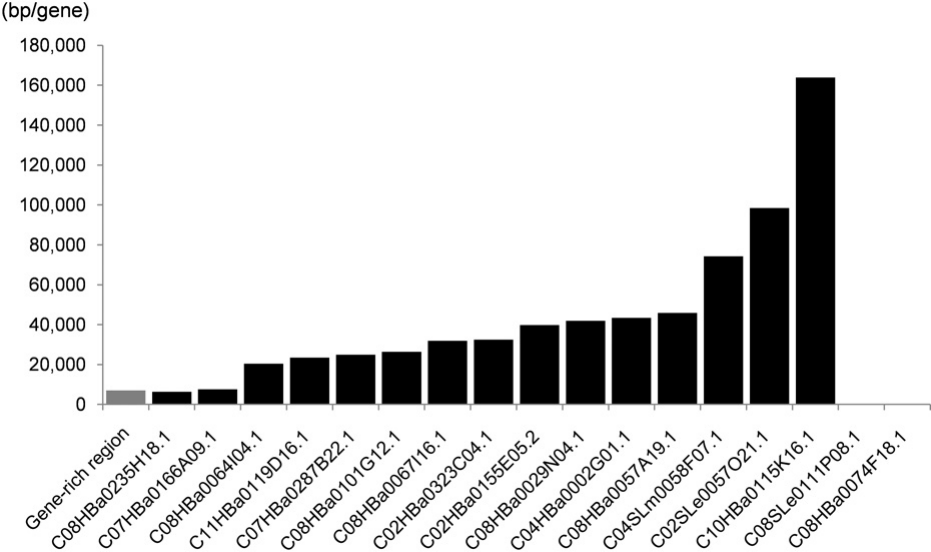
**The gene density of the tomato BAC sequences containing the Tat and Athila elements**. Gene-density of the seventeen tomato BAC sequences containing the Tat and Athila elements is presented. The gene density of the 'Gene-rich region' depicted by the gray column indicates the average gene density of the compared tomato gene-rich sequences. The gene number was counted with the exception of the transposable element genes. The gene density of the fifteen BAC sequences was lower than that of the gene-rich region by at least three times. The gene-density of the remaining two BAC sequences was similar to that of the gene-rich region. No genes were found in the C08SLe0111P08.1 and C08HBa0074F18.1.

To determine whether the tomato Tat and Athila elements are really accumulated in the heterochromatic regions in sequence-level, we investigated gene densities of the 17 tomato BAC sequences that contain the Tat and Athila elements (see Additional file 6). Two of the 17 tomato BAC sequences were gene-rich regions with a gene density similar to that of the compared tomato gene-rich sequences (Figure 7). However, the remaining 15 BAC sequences were gene-poor regions, in which the minimum gene density was about three times lower than that of the compared tomato gene-rich sequences. Considering that the tomato sequences are mainly from euchromatic regions, the accumulation of the tomato Tat and Athila elements shows a bias toward the heterochromatic regions. This result was consistent with the heterochromatin preferential distributions of the PCRT2 and PCRT3, indicating that the tomato Tat and Athila elements are accumulated in heterochromatic regions without the chromodomain.

### Proportion of the intact pepper *Ty3/Gypsy*-like elements in the genome

The proportion of the pepper *Ty3/Gypsy*-like elements in the genome was estimated using the intact LTR retrotransposons (see methods for detail) (Figure 6B). The proportion of the individual elements was broadly different according to the classified groups. The average proportion of the Tat elements was 1.28% but the Athila was 0.64%, suggesting more active accumulation of the Tat elements. The elements in the Del showed higher proportion than other classified groups in the genome as 2.01% of average proportion.

### Highly diversified features with similar lineage collections of *Ty1/Copia*-like elements

**Figure 8:**
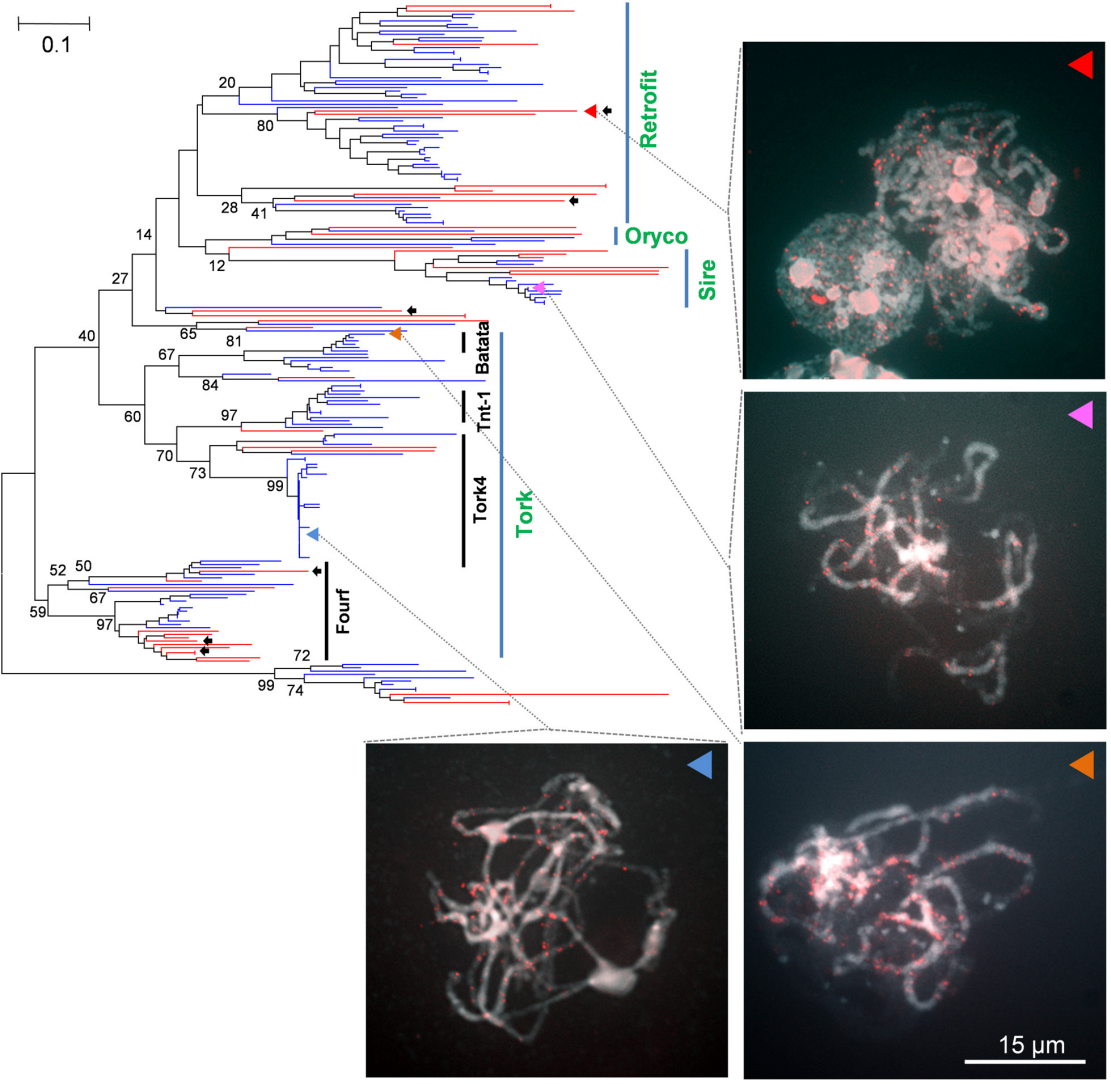
**Phylogenetic analysis of pepper and tomato *Ty1/Copia*-like elements**. Pepper and tomato reverse transcriptases (RT) of the *Ty1/Copia*-like elements were used in generating the phylogenetic tree. The pepper and tomato *Ty1/Copia*-like elements are depicted by red and blue lines, respectively. Classified types of each subgroup are depicted by green letters. The RTs used as FISH probes are marked with triangles (red, pink, orange, and blue triangles). The FISH result for of the probes is indicated by the dotted lines (see text for details). The black arrows indicate the RTs found from the compared pepper gene-rich sequences. The bootstrap values were produced by a replication of 1000.

The phylogenetic tree of *Ty1/Copia*-like elements presented highly diversified features that differed from those of *Ty3/Gypsy*-like elements. However they also showed similar lineage collections between pepper and tomato. By blast search against GyDB, four subgroups of the *Ty3/Copia*-like elements, Tork, Sire, Oryco, and Retrofit, were classified (Figure 8). The Tork was constituted with four subgroups that match to Fourf, Tork4, Tnt-1, and Batata. The six pepper *Ty1/Copia*-like elements found in the eight orthologous pepper BAC sequences are indicated by black arrows in Figure 8. These six elements belonged to diverse phylogenetic positions in the phylogenetic tree (Figure 8). One of the six pepper elements that belongs to the Retrofit was tested by FISH analysis, and the signals distributed randomly on the chromosomes (Figure 8; indicated by red triangle). The FISH signals of the tomato *Ty1/Copia*-like element that belongs to the Tork4 of the Tork subgroup were also observed in both brightly and darkly stained chromosome regions, indicating its distribution in heterochromatic and euchromatic regions (Figure 8; indicated by blue triangle). On the other hand, the FISH signals of tomato Batata element in the Tork subgroup and the elements in the Sire subgroup were observed mainly in the brightly stained chromosome regions, indicating a heterochromatin-preferential distribution (Figure 8; indicated by orange and pink triangle).

## Discussion

The results of the present study revealed that one of the important factors for the expansion of pepper euchromatic regions was the massive accumulation of the pepper Tat and Athila elements. In the Tat and Athila subgroups, the *Ty3/Gypsy*-like elements were found to be approximately two times more abundant in pepper than tomato. Considering that the pepper sequences used in this study were smaller than those of tomato in terms of total length (three-quarters of tomato) and in each contig length (Figure 1), the number of pepper Tat and Athila elements would further exceed that of tomato. Given that the tomato Tat and Athila elements preferentially accumulated in the heterochromatic regions (Figure 7), the higher copy number and random insertion of the pepper Tat and Athila elements suggests their important role in the expansion of pepper euchromatic regions.

According to the FISH analyses, the Del elements in both pepper and tomato genomes were identified as forming the pericentromeric heterochromatin blocks. Unlike the *Ty1/Copia*-like elements, the *Ty3/Gypsy*-like elements that constitute pericentromeric heterochromatin blocks are known to be selectively inserted into the heterochromatic regions in *A. thaliana *[30]. The existence of the chromodomain in both pepper and tomato Del intact elements can explain this insertion selectivity. The insertion site preferences of LTR retrotransposons have also been observed in other plant genomes, including conifers, and members of the genus *Helianthus *[31,32]. Although the number of Del elements is predominant in the phylogenetic tree, accumulation of the Del elements would have expanded the pericentromeric heterochromatin, not affecting euchromatin expansion in both species.

Pereira reported that the *Ty1/Copia*-like elements in *A. thaliana *were randomly inserted into the whole genome, after which they underwent purifying selection in euchromatic regions [30]. This resulted in the preferential accumulation of the *Ty1/Copia*-like elements in the pericentromeric heterochromatin blocks of *A. thaliana *genome. In the present study, a similar phenomenon was detected in the heterochromatin preferential accumulation of the tomato *Ty3/Gypsy*-like elements that belong to Tat and Athila (Figure 7). The absence of the chromodomain in the elements indicates its initial random insertions in the genome. However, the purifying selection may have been eliminated the tomato Tat and Athila elements from the tomato euchromatic regions, resulting in their preferential accumulation in the heterochromatic regions.

The comparative analysis of the LTR retrotransposons in the present study revealed a similar collection of lineages in both *Ty3/Gypsy*-like and *Ty1/Copia*-like elements. Given that the LTR retrotransposons of the same lineage have similar characteristics, both genomes would have accumulated the *Ty1/Copia*-like elements and *Ty3/Gypsy*-like elements of the Tat and Athila in their euchromatic regions. However, the copy number of the elements in the tomato euchromatic regions may have been reduced due to the purifying selection, which could have resulted in the lower accumulation of the LTR retrotransposons in the tomato euchromatic regions than in those of pepper.

The number of pepper Del elements corresponded to twice the number of tomato Del elements (256 in pepper and 122 in tomato). This difference was partially due to the euchromatin selective sequencing of the tomato genome project. Although the pepper BAC clones were selected by the labelled cDNAs of mRNAs, our bioinformatics survey suggested that a large portion of pepper BAC clones contained heterochromatic regions. This phenomenon can be caused by contamination with the transcripts of repetitive elements, such as retrotransposons, during the selection of the BAC clones.

The expansion of genome sizes through the accumulation of LTR retrotransposons is well documented among flowering plants [30,33-35]. Based on the results of the present study, the expansion of the pepper genome is also due to the accumulation of LTR retrotransposons. A similar comparative analysis of repetitive elements in close species was carried out between *A. thaliana *and *Brassica oleracea *by Zang *et al. *[36]. Zang *et al. *reported that the large size of the *B. oleracea *genome is accounted by the higher copy number of each type of transposable elements within a similar collection of lineages, explaining the overall genome expansion [36]. However, the gene densities of both genomes were 4.5 kb/gene in *A. thaliana *and 6.6 kb/gene in *B. oleracea *[37], indicating that the euchromatic regions of both genomes are highly gene-rich, as is the case in tomato. In contrast with *B. oleracea*, pepper has an expanded euchromatin structure, and the present results explained the expansion of the euchromatic regions. Hence, the comparison of the pepper and tomato genomes can provide new insights into the expansion of euchromatic regions by the accumulation of repetitive elements.

## Conclusions

The results of the present study show that the *Ty3/Gypsy*-like elements in the Tat and Athila play an important role in the expansion of the pepper euchromatic regions. The genic regions of pepper and tomato were found to be well conserved with regards to gene order and content. However, the euchromatic regions in pepper were expanded to twice the size of those in tomato, mainly due to the insertion of LTR retrotransposons. The LTR retrotransposons in the pepper euchromatic regions may also explain why the pepper euchromatic regions look like intermixed with the heterochromatin structures.

## Methods

### Sequencing of the pepper and tomato BAC clones

The selection of pepper BAC clones for the comparison of the orthologous gene-rich regions was performed as described in this section: seven tomato BAC sequences that are distributed on chromosome 2 were chosen. The tomato sequences were used as queries in a BLASTN search of the pepper EST database to find orthologous pepper ESTs. Two or three pepper ESTs that were orthologous to sequences about 30 kb apart in each tomato BAC clone were used as probes. The probes were labelled via PCR amplification using specific primers and ^32^P-labeled dCTP. Seven or eight labelled probes were pooled, and the Southern hybridizations were carried out on pepper BAC library filters. The filters were sequentially washed in 2 X SSC for 60 min, 1 X SSC for 60 min, and 0.5 X SSC 60 min. The positive clones were confirmed by colony PCR using the same primers. The pepper BAC clones that showed positive PCR results were used for sequencing. Each BAC clone was fully sequenced and analyzed by NICEM (http://nicem.snu.ac.kr/) using the ABI 3730xl system (Applied Biosystems Inc [ABI], Foster City, CA). From each pepper BAC clone, a shotgun sequencing library was constructed using the pUC118 vector with an average insert size of 3-5 kb. BigDye Terminator chemistry version 3.1 (ABI) was used for the sequencing reactions. All of the sequences were analyzed by Phred/Pharp/Consed processing [38]. Base-calling and assembling of the sequences were carried out using the Phred/Phrap software. The Phred scores of the sequences were 30 or higher. The assembled sequences were edited using Consed software. Sequence editing for consensus contig formation was carried out using the Sequencher 4.1.5 (Gene codes Corp., Ann Arbor, USA). The tomato BAC sequences were generated by the same method as part of the International Tomato Sequencing Project of Korea [20].

A total of 1,235 pepper BAC clones were additionally selected for next-generation sequencing using 454 GS FLX-Titanium (454 Life Science, Roche). Each BAC clone DNA was manually extracted and normalized. The normalized BAC clone DNAs were pooled into 125 clones per a reaction channel of 454 GS FLX-Ti. Each sequencing reaction of 454 GS FLX-Ti was divided into two. The sequencing procedures for the 454 GS FLX-Ti were carried out using manufacturer-supplied protocols and reagents. The sequences were assembled by Newbler 2.0.1. The average coverage of the contigs was 18.22×. Among the assembled contigs, the contigs longer than 30 kb were used in the analyses.

### Gene prediction and comparative analysis

For accurate gene structure analysis, we predicted genes using three steps as follows: (1) Genes were predicted by FGENESH using a trained data set from tomato [39]. (2) The predicted genes were confirmed by BLASTP searches of the GenBank database (http://www.ncbi.nlm.nih.gov/) using the protein sequences of the predicted genes as queries. Among the predicted genes, those with scores greater than 100 and e-values less than e-20 were used in the next step. (3) Among the BLASTP results for each predicted gene, the protein sequence that had the highest score was chosen as a reference, and the gene models were predicted again by FGENESH+ using the trained data set from tomato. These results were used as gene models for the pepper and tomato sequences. The visualization of compared orthologous sequences was carried out using GATA [40] with minimum bits of 30 and maximum bits of 35.6. The repeat sequences were found by BLAST search in the Repbase repeat masking (http://girinst.org/censor/index.php) [24,25].

### Phylogenetic analysis of LTR retrotransposons

The RTs were found by Hmmer 2.1 [41] using the RTs reported in the Pfam database (http://pfam.sanger.ac.uk/, Accession No. PF00078 and PF07727) as a training set. The super family of the RTs was determined by the Rebase repeat masking. The RTs were confirmed by BLASTP searches in the GenBank database, and the RTs with a score over 200 were used in the analyses. The RTs containing any frame shift mutations or deletions were manually deleted in the alignment. Intact LTR retrotransposons were predicted using LTR_FINDER [42] with the default settings or by manual inspection using DOT-PLOT analysis. These analyses were conducted in the environment of the Comparative Fungal Genomics Platform (CFGP; http://cfgp.snu.ac.kr/) [43].

The phylogenetic trees were generated using the MEGA 4.0 software [44]. The alignments were carried out using ClustalW of MEGA 4.0 with the default settings (see Additional file 7, 8 and 9). The aligned RT sequences were used for generating the phylogenetic trees. The Poisson correction model and Neighbor-Joining method were used, and the phylogeny test was carried out by bootstrapping with 1,000 replications.

### Chromodomain search and proportion calculation of the intact LTR retrotransposon in the genome

The chromodomains were found by BLASTX search of the intact LTR retrotransposons with the chromodomain proteins in the Pfam database (PF00385). The chromodomains found in the intact LTR retrotransposons were used again in finding the chromodomains in the other sequences. The intact LTR retrotransposons that have no chromodomain were confirmed again by the conserved domain search service in the NCBI (http://www.ncbi.nlm.nih.gov/Structure/cdd/wrpsb.cgi).

The proportions of the individual intact LTR retrotransposons in the pepper genome were calculated using the 90.8 Mbp of the assembled sequence and 3.2 Mbp of additional BAC sequences of pepper. By BLASTN search against the total 94 Mbp pepper sequence, total matched sequence length of each intact LTR retrotransposon was calculated. The threshold e-value of the search was e-5. The total matched sequence length of the individual intact LTR retrotransposon was divided by the 94 Mbp of pepper sequence size.

### FISH analysis

The FISH probes for analyzing the LTR retrotransposons were produced by PCR amplification using the primer sets listed in Additional file 10 online. Pachytene chromosomes of tomato (*Lycopersicon esculentum *cv. Micro-Tom) were prepared according to the methods of Koo et al. [45]. Metaphase and pachytene chromosomes of pepper (*Capsicum annuum *cv. CM334) were prepared as described by Kwon et al. [46]. All probes were labelled with biotin 16-dUTP or digoxygenin 11-dUTP by nick translation as described by the manufacturer's protocol (Roche, Germany). The FISH experiments for tomato and pepper were performed according to the methods described by Koo et al. [45] and Kwon et al. [46], respectively. The hybridization solutions contained 50% formamide (w/v), 10% dextran sulfate (w/v), 5 ng/μl salmon sperm DNA, and 20 ng of each probe in 2 X SSC. The probes were detected using fluorescein avidin DCS (Roche, Germany) and rhodamine anti-digoxygenin (Roche, Germany). Pachytene chromosomes were counterstained with DAPI (1 mg/ml) in Vectashield antifade (Vector Laboratories). All images were captured and analyzed using the DeltaVision imaging system and associated software (Applied Precision, USA) with a cool SNAP CCD camera at NICEM. All images were improved for optimal brightness and contrast using Adobe Photoshop.

## Abbreviations

BAC: bacterial artificial chromosome, EST: expressed sequence tag, FISH: fluorescence *in situ *hybridization, LTR: long terminal repeat, NGS: next-generation sequencing, RT: reverse transcriptase

## Authors' contributions

MP designed this study, carried out the overall sequence analysis, and wrote the draft manuscript. SJ generated the tomato sequence data and helped the manuscript revise. JKK carried out the fluorescence *in situ *hybridization. JP assembled the pepper sequence data and helped sequence analysis. JHA carried out pepper BAC clone sequencing. SK participated in the pepper sequence assembly. JP, YHL, TJY, CGH, BCK, and BDK helped the manuscript revise. DC conceived the study, helped to draft the manuscript, and organized the sequencing of pepper and tomato BAC sequences. All authors read and approved the final manuscript.

## Supplementary Material

Additional file 1 **The compared BAC sequence sizes and GenBank accession numbers**. Click here for file

Additional file 2 **Information about predicted genes of the compared sequences**. Click here for file

Additional file 3 **Information about duplicated genes**. Click here for file

Additional file 4 **Sequence information of the 72 intact *Ty3/Gypsy*-like elements**. Click here for file

Additional file 5 **List of chromodomains isolated from intact pepper and tomato LTR retrotransposons**. Click here for file

Additional file 6 **Information about predicted genes in the 17 tomato BAC sequences containing Tat and Athila elements**. Click here for file

Additional file 7 **Alignment of reverse transcriptases of *Ty3/Gypsy*-like elements**. Click here for file

Additional file 8 **Alignment of reverse transcriptases isolated from intact pepper and tomato LTR retrotransposons**. Click here for file

Additional file 9 **Alignment of reverse transcriptases of *Ty1/Copia*-like elements**. Click here for file

Additional file 10 **List of PCR primers used to produce the FISH probes**. Click here for file
